# Protected Percutaneous Coronary Intervention (PCI) of Cabrol Type Anastomosis of Saphenous Vein Graft (SVG) to the Left Main Coronary Artery (LMCA)

**DOI:** 10.7759/cureus.8829

**Published:** 2020-06-25

**Authors:** Sabah Siddiqui, Sergey Ayzenberg, Nitin Sabharwal, Robert Frankel, Jacob Shani

**Affiliations:** 1 Cardiology, Maimonides Medical Center, Brooklyn, USA; 2 Cardiology, James J. Peters Va Medical Center, Bronx, USA; 3 Cardiology, Tulane University School of Medicine, New Orleans, USA

**Keywords:** cabrol type anastomosis, impella cp, hemodynamics, physiology, pulsatility

## Abstract

The use of percutaneous left ventricular assist devices (VAD) may minimize the risk of hemodynamic compromise during such high-risk percutaneous coronary intervention (PCI) and allow complete revascularization, thus improving outcomes. A good understanding of cardiac hemodynamics is essential in making informed decisions during such cases. A 61-year-old male with an extensive surgical cardiac history including a modified Cabrol type anastomosis with saphenous vein (SVG) conduits to two coronary arteries presented to our hospital with severe substernal chest discomfort and was noted to have diffuse ST depressions along with subtle ST elevations in lead aVR suggestive of diffuse sub-endocardial ischemia. Diagnostic coronary angiography revealed significant stenosis in the Cabrol type SVG grafts and we opted for a protected PCI using Impella (Abiomed, Danvers, MA) support. A significant drop in the blood pressure was noted and despite trouble-shooting, the Impella arterial line tracing remained minimally pulsatile.​ A comprehensive understanding of circulatory support physiology was ultimately crucial in making an informed decision for a successful PCI outcome.

## Introduction

Percutaneous catheter-based transvalvular devices for temporary use such as Impella CP help pump blood from the left ventricle (LV) to the arterial system [1]. The use of percutaneous left ventricular assist devices (VAD) may minimize the risk of hemodynamic compromise during such high-risk percutaneous coronary intervention (PCI) and allow complete revascularization, thus improving outcomes [2]. Hemodynamics may change very rapidly during such high-risk PCI cases. It is therefore critical to understand the primary hemodynamic effects of a device and the expected effects on pressures and flow in the absence of any change in native heart or vascular properties. In addition, it is also important to observe the overall net hemodynamic effects after accounting for the impact of secondary modulating factors [3]. This is crucial in making informed decisions during PCI with mechanical support.

## Case presentation

A 61-year-old male with an extensive surgical cardiac history, including aortic stenosis status post aortic valve replacement and mitral valve repair in 2007, with no prior coronary artery disease, chronic kidney disease, diabetes, status post two redo-sternotomies due to prosthetic valve endocarditis requiring bioprosthetic aortic valve replacement in 2013, mitral valve endocarditis in 2017 treated medically with intravenous antibiotics and on lifelong oral doxycycline, presented to Maimonides Medical Center, NY on August 2018, with shortness of breath on exertion. ​Physical examination was significant for a grade 3/4 diastolic murmur on auscultation. Lab work was significant for a B-type natriuretic peptide (BNP) of 653 pg/ml (normal < 100 pg/mL) and echocardiography revealed significant aortic para-valvular leak. ​Surgical aortic valve replacement was offered, and prior to the redo-sternotomy, coronary angiogram showed no significant coronary artery disease.​ During surgical exploration, due to concern for aortic root abscess, in addition to replacement of the aortic valve, he also required aortic root repair with a homograft, and modified Cabrol type anastomosis with saphenous vein (SVG) conduits to the left main coronary artery (LMCA) and the right coronary artery (RCA).

**Figure 1:**
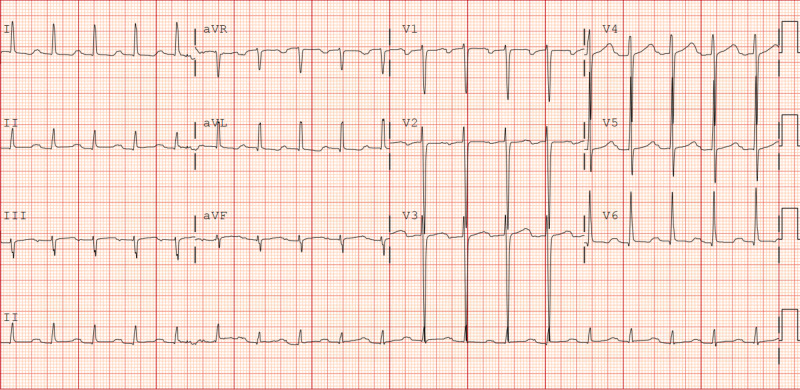
Baseline EKG

**Figure 2:**
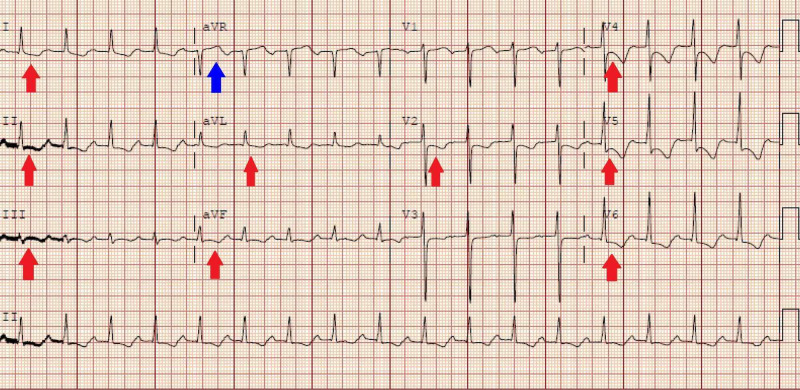
EKG showing diffuse ST depressions (Red arrows) along with subtle ST elevations in lead aVR (Blue arrow) suggestive of diffuse sub-endocardial ischemia

He presented to our hospital a few months later with intermittent substernal chest discomfort 7 out of 10 in intensity. Compared to his baseline EKG (Figure 1), he was intermittently noted to have diffuse ST depressions along with subtle ST elevations in lead aVR suggestive of diffuse sub-endocardial ischemia (Figure 2), and he ruled in for a non-ST elevation myocardial infarction (NSTEMI) with a max troponin of 3 (normal <0.04 ng/ml).

**Figure 3:**
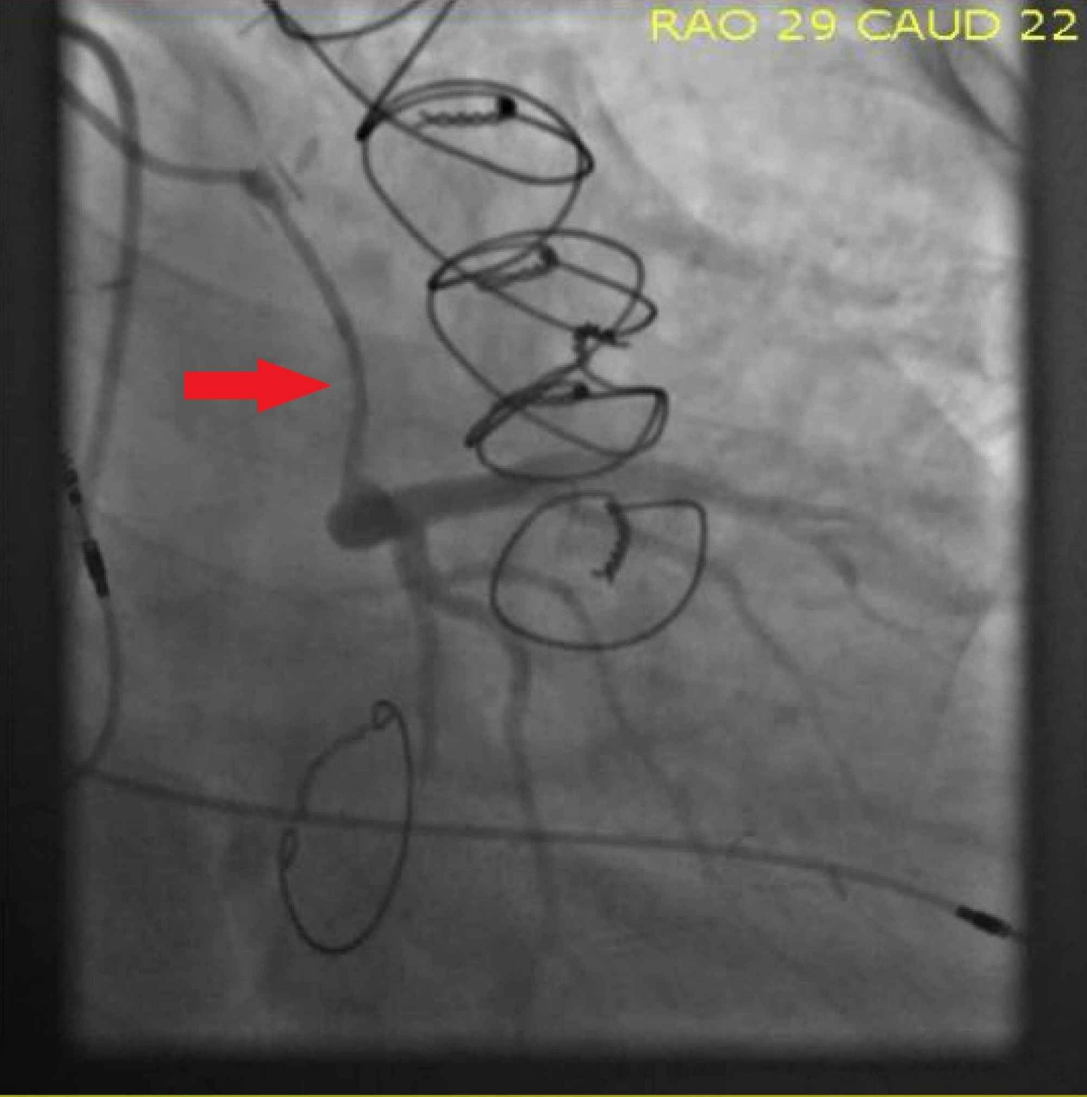
Diagnostic coronary angiography revealed a 90% stenosis of the Cabrol type SVG graft to the LMCA (Red arrow) with TIMI 3 flow SVG: Saphenous vein graft; LMCA: Left main coronary artery.

**Figure 4:**
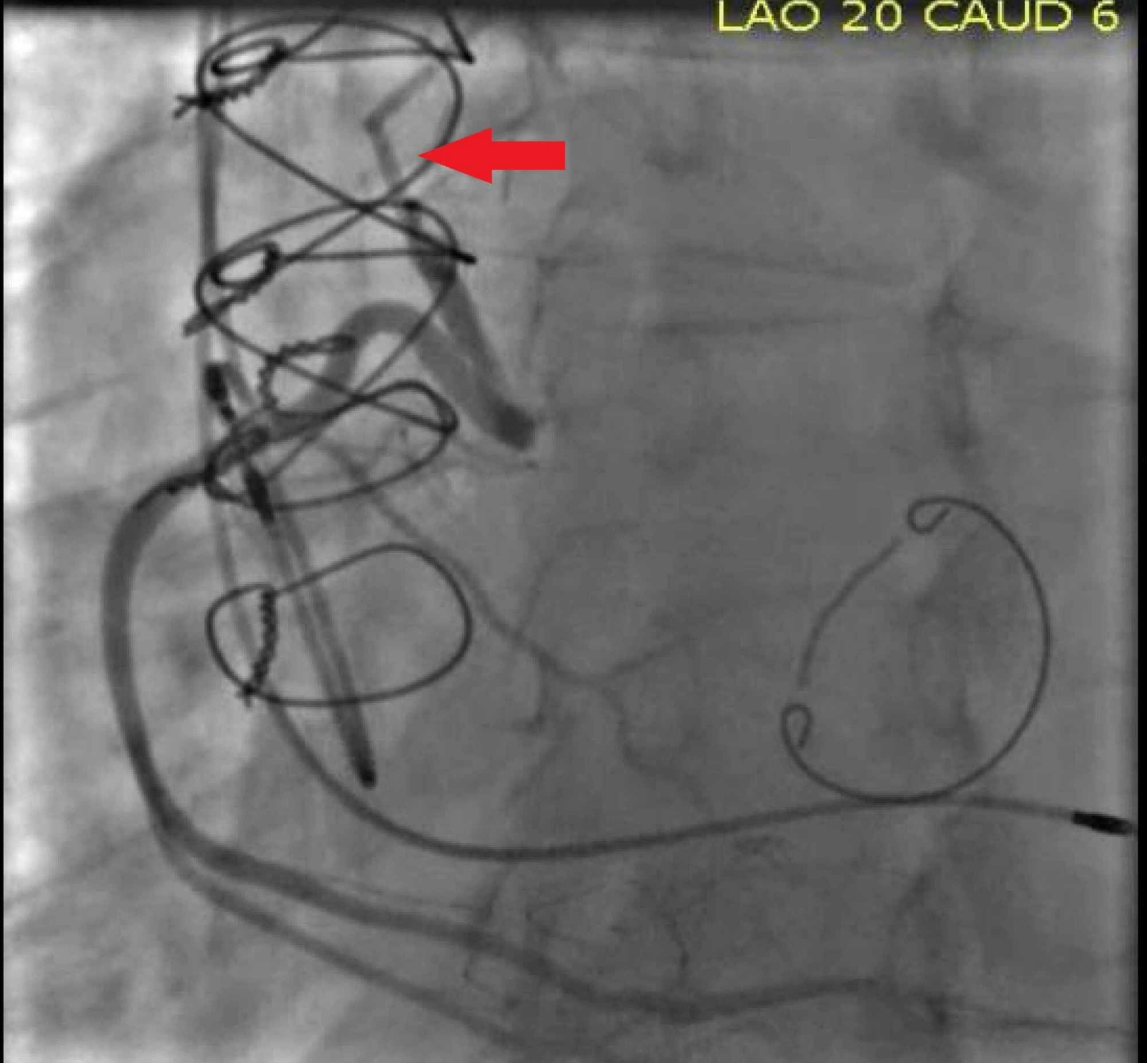
Diagnostic coronary angiography revealed an 80% ostial stenosis of the Cabrol type SVG graft to the RCA (Red arrow) SVG: Saphenous vein graft; RCA: Right coronary artery.

Diagnostic coronary angiography revealed a 90% stenosis of the Cabrol type SVG graft to the LMCA (Figure 3) with TIMI 3 flow and an 80% ostial stenosis of the Cabrol type SVG graft to the RCA (Figure 4).​ After heart team discussion, due to his high surgical risk as a result of multiple sternotomies, we opted for a protected percutaneous coronary intervention (PCI) using Impella (Abiomed, Danvers, MA) support [4].

**Figure 5:**
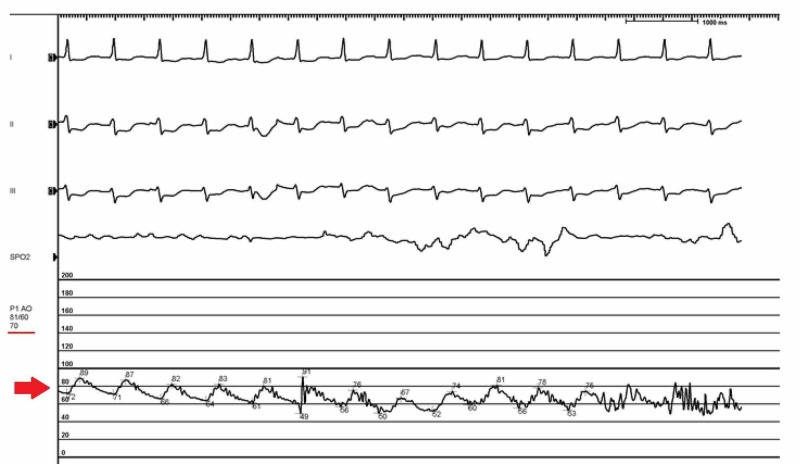
Arterial line tracing remained minimally pulsatile with a blood pressure of 81/60 (67) mm Hg (Red arrow)

The patient was brought back for this procedure and bilateral femoral arteries were accessed, and initial aortic opening pressure was recorded as 120/74 (89) mm Hg. Left femoral artery sheath was upsized to 14F and Impella was inserted under fluoroscopic guidance. As we positioned the Impella into the LV cavity, a significant drop in the blood pressure was noted. ​Arterial tracing from the right femoral access revealed a blood pressure of 68/38 (51). After administration of fluids, vasopressors, and increasing Impella settings to P8, the mean arterial pressure (MAP) increased to 70 mm Hg, however, the arterial line tracing remained minimally pulsatile with a blood pressure of 81/60 (67) mm Hg (Figure 5). ​The patient remained asymptomatic during this period.

**Figure 6:**
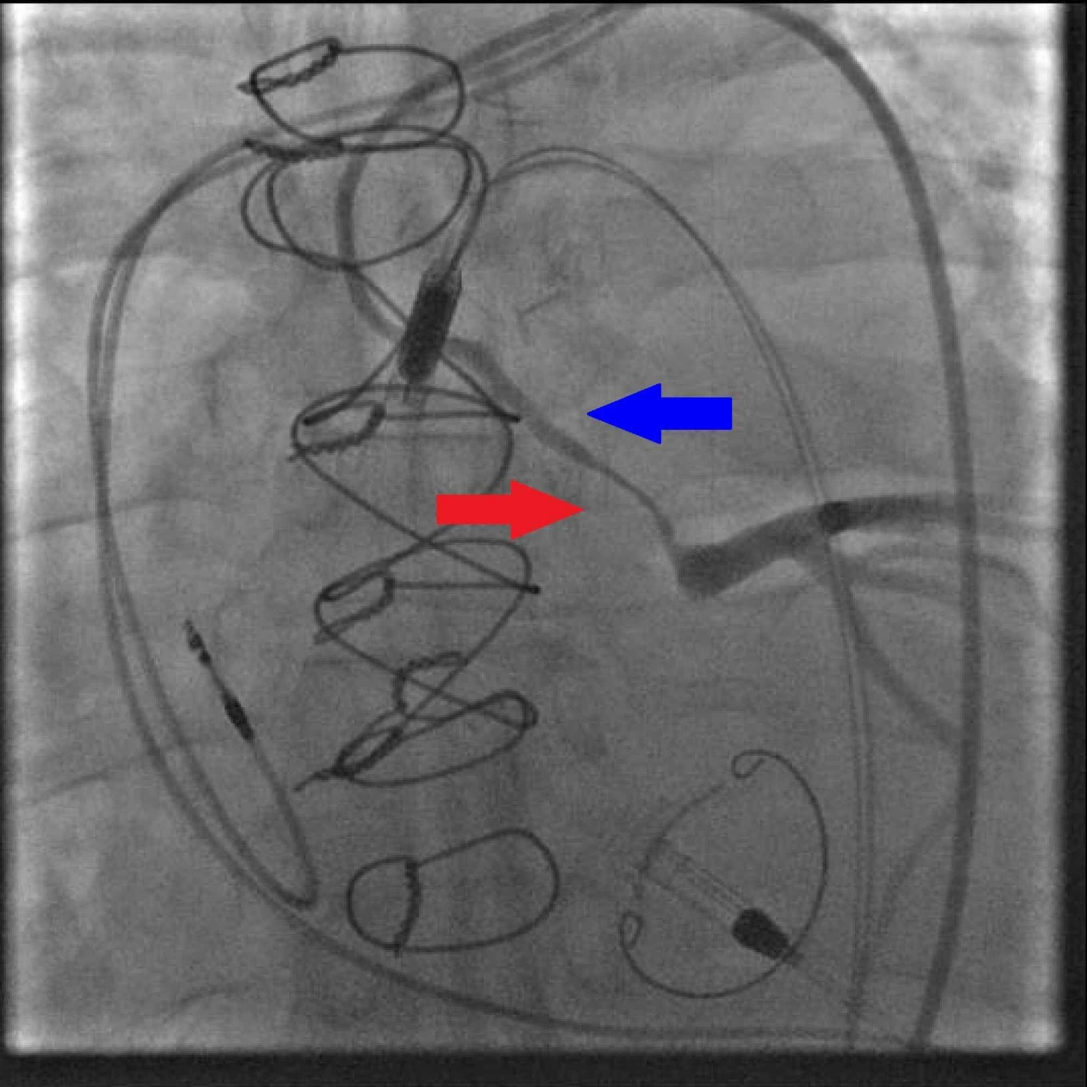
Coronary angiography of the Cabrol graft showed the previously known stenotic lesion (Blue arrow) with intermittent vasospasm (Red arrow) resulting in diffuse ischemia and as a result, reduced contractility of the native myocardium

We assessed the Impella console for suction alarms and device malfunction. However, no alarms were noted, and a similar mean arterial pressure along with minimal pulsatility was noted on the arterial tracing from the Impella console.​ Due to the true and unexpected decrease in pulsatility confirmed via simultaneous measurement of the right femoral access as well as the Impella waveform, we suspected that although the Impella was providing adequate perfusion, there was loss of intrinsic contractility of the myocardium, likely as a consequence of reduced flow through the SVG conduit to the LMCA.​ Angiography of the Cabrol graft showed the previously known stenotic lesion with intermittent vasospasm resulting in diffuse ischemia and as a result, reduced contractility of the native myocardium (Figure 6).

**Figure 7:**
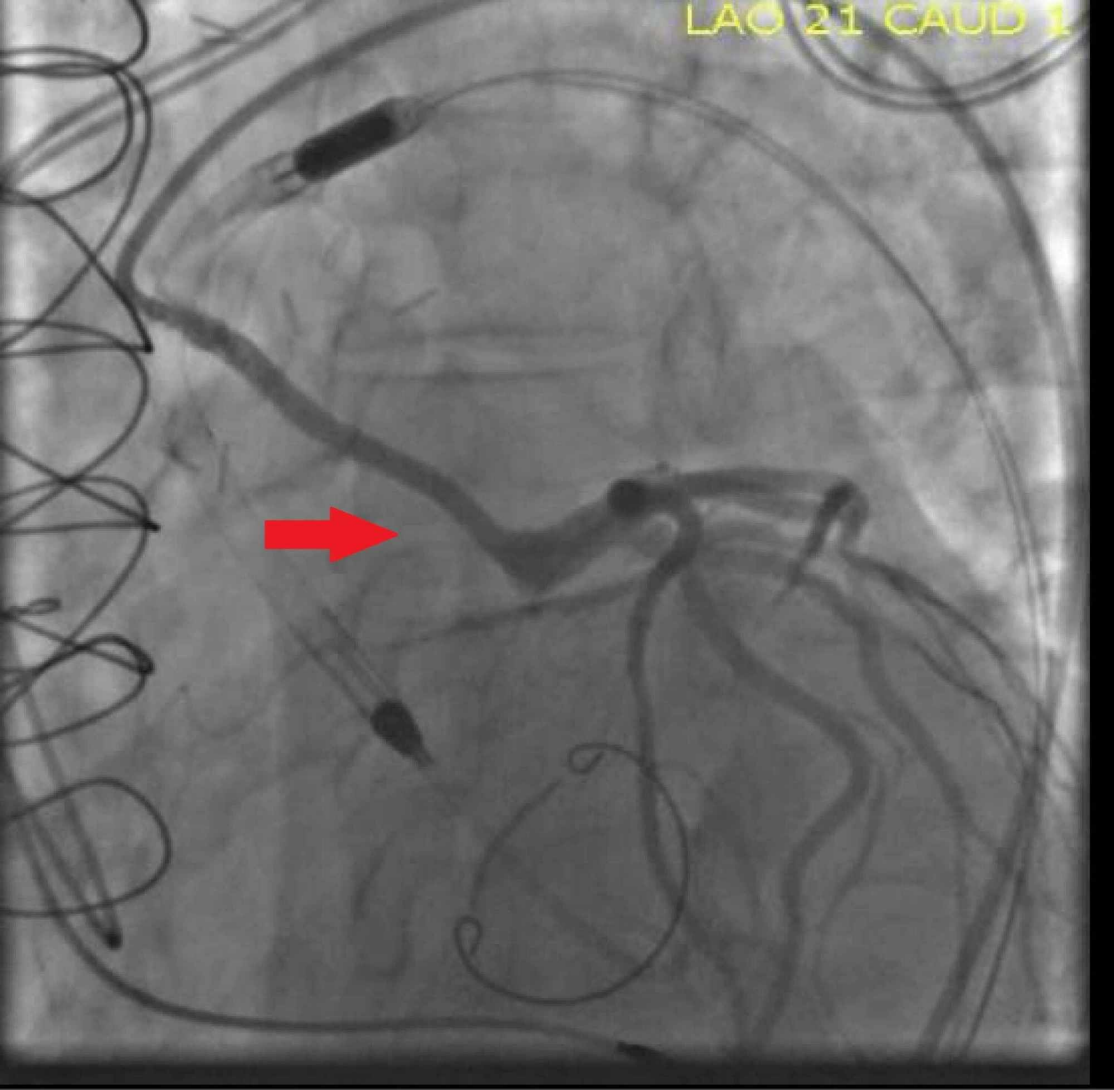
Coronary angiography showing direct stent of the SVG to LMCA with a 3 x 38 mm Promus premier stent in the body of the graft, and an overlapping 3.5 x 12 mm Promus premier stent covering the ostium of the SVG graft with an excellent angiographic result (Red arrow) SVG: Saphenous vein graft; LMCA: Left main coronary artery.

​We proceeded with the intervention despite the minimal pulsatility in view of normal mean arterial pressure and adequate cerebral perfusion. We directly stented the SVG to LMCA with a 3 x 38 mm Promus premier stent in the body of the graft, and an overlapping 3.5 x 12 mm Promus premier stent covering the ostium of the SVG graft with an excellent angiographic result (Figure 7).

**Figure 8:**
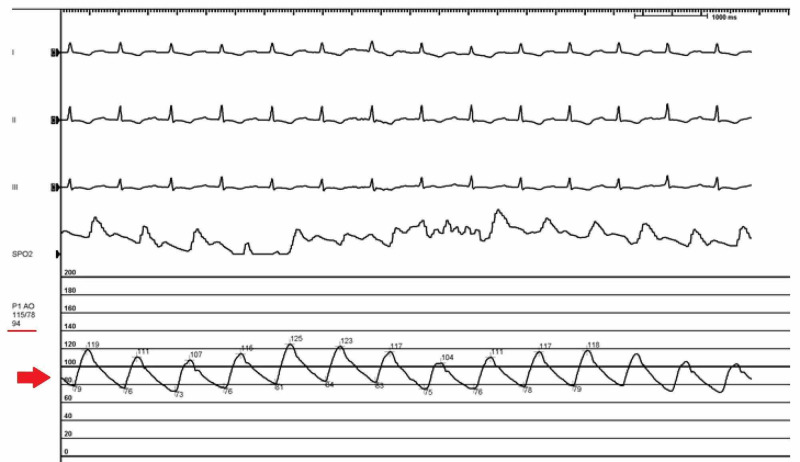
Repeat blood pressure was recorded as 115/78 (94) mm Hg (Red arrow)

A pulsatile waveform was noted immediately after the intervention.​ Increased pulse pressure was noted on the arterial waveforms from both the right femoral artery, as well as the Impella. ​Repeat blood pressure was recorded as 115/78 (94) mm Hg (Figure 8, Table 1).

**Table 1:** Blood pressure, mean arterial pressure, and heart rate recordings throughout the case AO: Aortic site; LV: Left ventricle; MAP: Mean arterial pressure P8: Impella flow level; maximum support level

Timing	Site	Systolic BP	Diastolic BP	MAP	Heart Rate
Opening pressure	AO	120	74	89	90
After Impella in position in LV	AO	68	38	51	92
After administration of fluids, vasopressors and P8 Impella setting	AO	81	60	70	94
Repeat Blood pressure	AO	94	71	80	99
Immediately post intervention	AO	115	78	94	87
Repeat Blood pressure	AO	112	75	90	88
Closing pressure	AO	127	85	100	90

The patient tolerated the procedure well and was successfully weaned off the Impella after the PCI and discharged.​ He later underwent staged PCI of the Cabrol type SVG graft to the RCA.​ Currently, he is followed as an outpatient and remains asymptomatic.

## Discussion

Pulsatility is generated by the native heart as it cycles through systole and diastole [3]. ​The use of transvalvular VAD generates a mean flow throughout the cardiac cycle including isovolumetric contraction and relaxation [3].​ The Impella CP (Abiomed) is a circulatory support system that is positioned across the aortic valve under radiographic or echocardiographic guidance [5]. It is a 14 Fr size micro-axial blood pump, mounted on a 9 Fr catheter, and it aspirates blood from the left ventricle into the ascending aorta and works independently of the cardiac rhythm. With its maximal speed of 46,000 revolutions per minute (rpm), the device enhances the blood flow from the LV to the aorta by a maximum of 3.3 to 3.5 l/min in clinical conditions [6]. Impella-mediated LV unloading reduces end-diastolic wall stress, improves diastolic compliance, increases aortic and intracoronary pressure and coronary flow velocity reserve, and stimulates a decrease in coronary microvascular resistance [7,8]. The use of percutaneous left ventricular assist devices may minimize the risk of hemodynamic compromise during such high-risk PCI and allow complete revascularization, thus improving outcomes [8].

**Figure 9:**
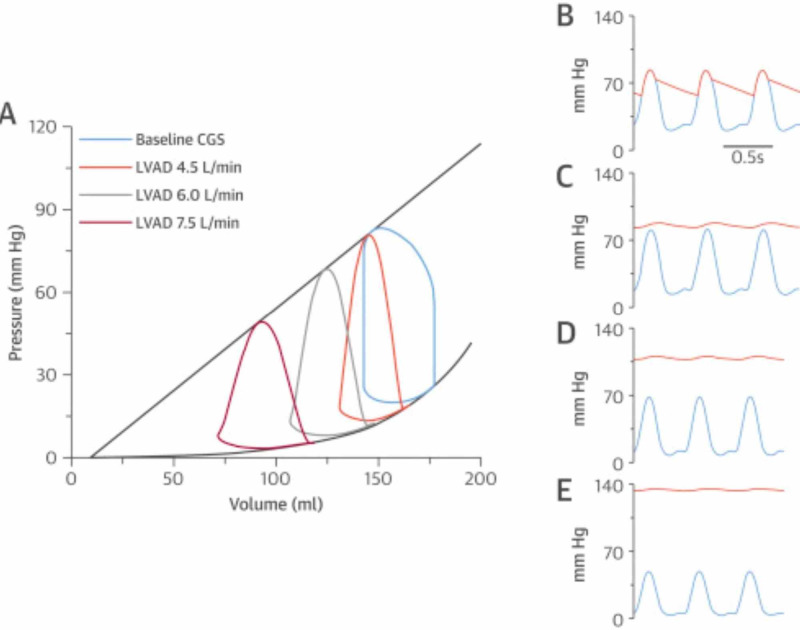
Shows flow-dependent changes of the pressure-volume loop with LV-to-aortic pumping Uncoupling between aortic and peak LV pressure generation as seen at baseline (B), 4.5 l/min (C), 6.0 l/min (D) and 7.5 l/min (E). Republished with permission from [3].

Pulsatile waveform can still be present with the use of VADs due to adequate pulsatility generated by the myocardium to open the aortic valve, as well as due to a fluctuating pump head pressure (∆P) between systole and diastole [9]. However, when there is a significant compromise to coronary flow, as we suspect had occurred during our intervention, mechanical contraction of the heart may be severely diminished resulting in a significant loss of pulsatility and in extreme cases, only a mean pressure tracing maybe noted on the arterial line [10]. ​Loss of or diminished native pulsatility in the setting of a stable MAP and adequate perfusion to the patient may indicate a greater degree of hemodynamic support from the Impella and not device malfunction [3]. The hemodynamics of mechanical circulatory support (MCS) is most clearly depicted in terms of pressure-volume analysis [11]. Figure 9A shows flow-dependent changes of the pressure-volume loop with LV-to-aortic pumping. As the pump flow rate increases, the LV becomes increasingly unloaded. The loop becomes triangular and shifts progressively leftward showing a decrease in peak LV pressure generation, pressure-volume area, and myocardial oxygen consumption. As arterial pressure increases, it becomes increasingly dissociated from the peak LV pressure. With increased flow, there are greater degrees of LV unloading and uncoupling between aortic and peak LV pressure generation as seen at baseline (Figure 9B), 4.5 l/min (Figure 9C), 6.0 l/min (Figure 9D) and 7.5 l/min (Figure 9E) [3]. Loss of or diminished native pulsatility in the setting of a stable MAP and adequate perfusion to the patient may indicate a greater degree of hemodynamic support from the Impella and not device malfunction [3].

## Conclusions

Herein, we presented a case of a patient with an extensive surgical cardiac history who presented with an NSTEMI and chest pain who underwent complex high-risk PCI. Due to his high surgical risk as a result of multiple sternotomies, we opted for a protected PCI using Impella support. However, during the case, as a result of dynamic changes, we had to go through the understanding of cardiac hemodynamics and troubleshooting with the Impella CP. Therefore, a comprehensive understanding of circulatory support physiology is crucial for making informed decisions during PCI with mechanical circulatory support.​
